# Supplementary material for: Altered glutamate homeostasis in paramagnetic rim lesions of patients with multiple sclerosis
Source: Imaging Neurosci (Camb). 2026 Nov 10;4:IMAG.a.1406. doi: 10.1162/IMAG.a.1406 (PMC13754067; doi:10.1162/IMAG.a.1406)
Supplement: Supplementary file 1 [file IMAG.a.1406_supp.pdf]

Supplemental Material

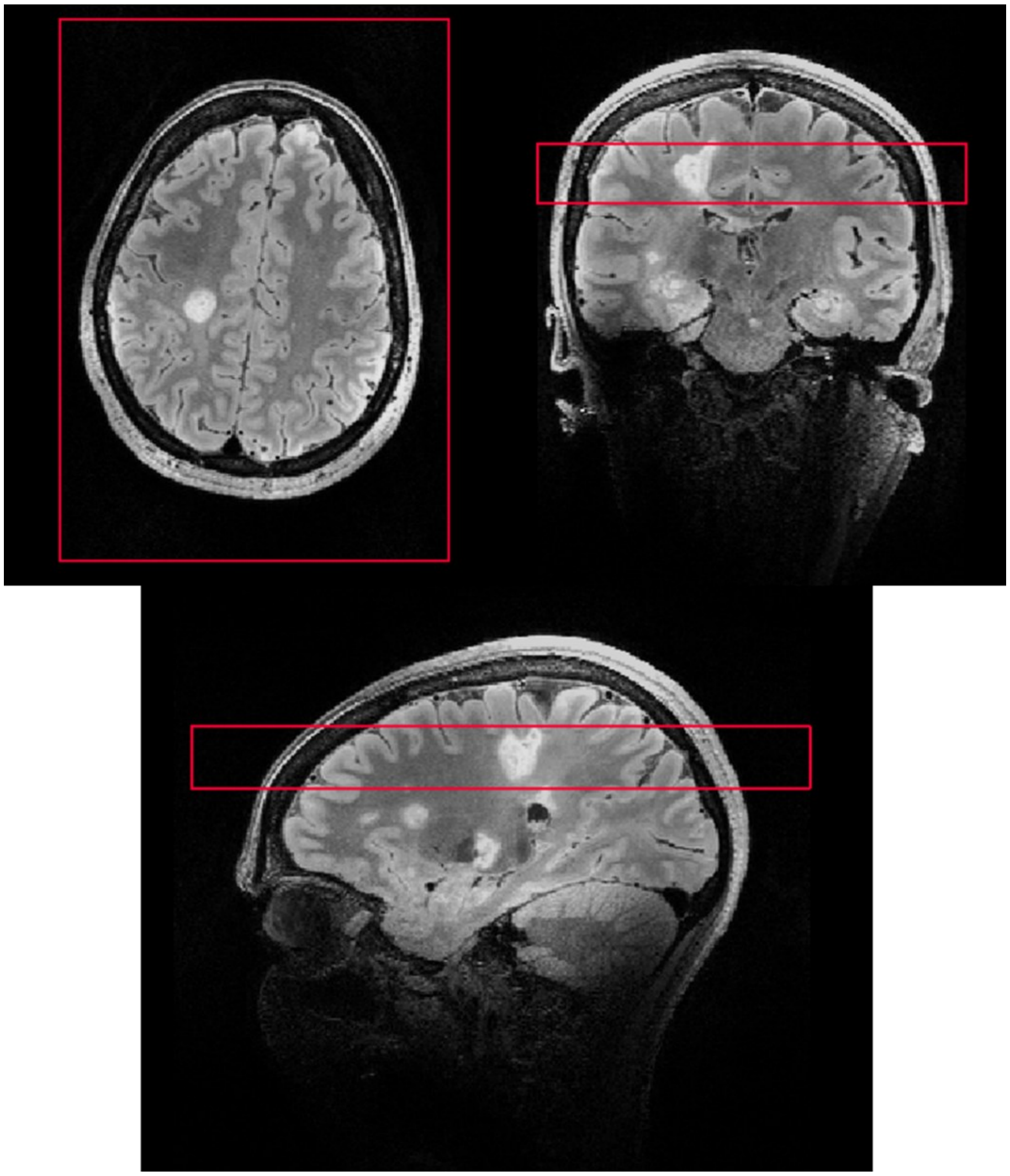

Supplemental Figure 1. A set of representative example images of the brain slab volume in which axial (top left), coronal (top right), and sagittal (bottom center) FLAIR images can be seen with an overlaid red outline corresponding to the size and location of the 20 mm GluCEST “brain slab” acquisition.

Supplemental Table 1. Summarized groupwise T_1 values, QSM values, and GluCEST contrast values

		T_1 value (ms)	QSM (ppb)	GluCEST (%)
**Whole Brain**	**Control**	1593.9±75.4	-	7.97±0.85
	**MS**	1688.6±114.5	0.39±0.48	7.50±1.01
**Gray Matter**	**Control**	1770.3±19.9	-	9.47±0.68
	**MS**	1773±12.5	2.46±0.95	8.94±0.94
**White Matter**	**Control**	1155.9±17.1	-	7.27±1.18
	**MS**	1173.4±22.2	-2.77±0.94	7.12±1.09
**Non PRL**		1609.2±365.6	-0.68±3.25	7.27±1.36
**PRL**		1653.0±280.9	4.26±7.27	8.05±0.97

Summarized average (standard deviation) values for T_1 and GluCEST contrast values across all segmented tissue types for both MS and healthy control data.

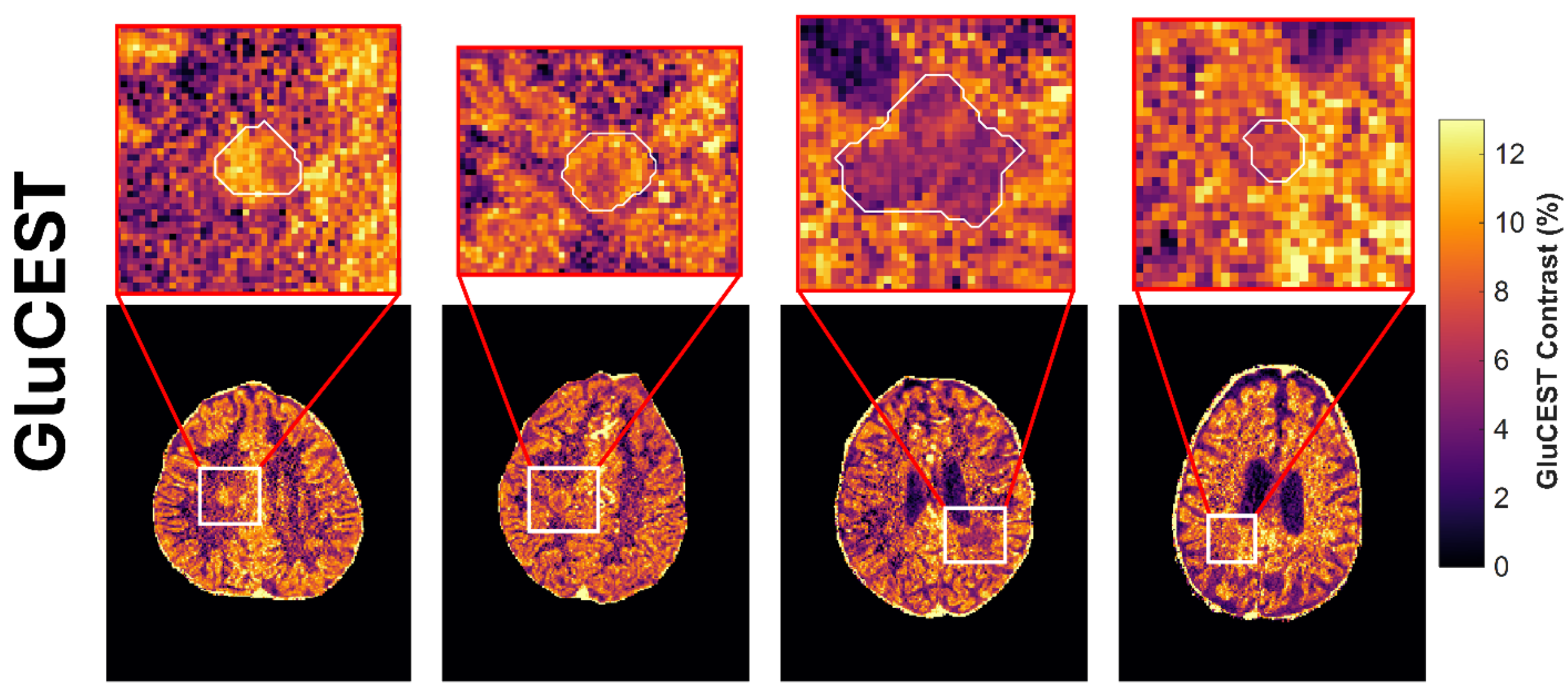

Supplemental Figure 2. An annotated set of GluCEST images from figure 2 showing the outline (white) where the PRLs and non-PRLs are relative to the surrounding tissue.

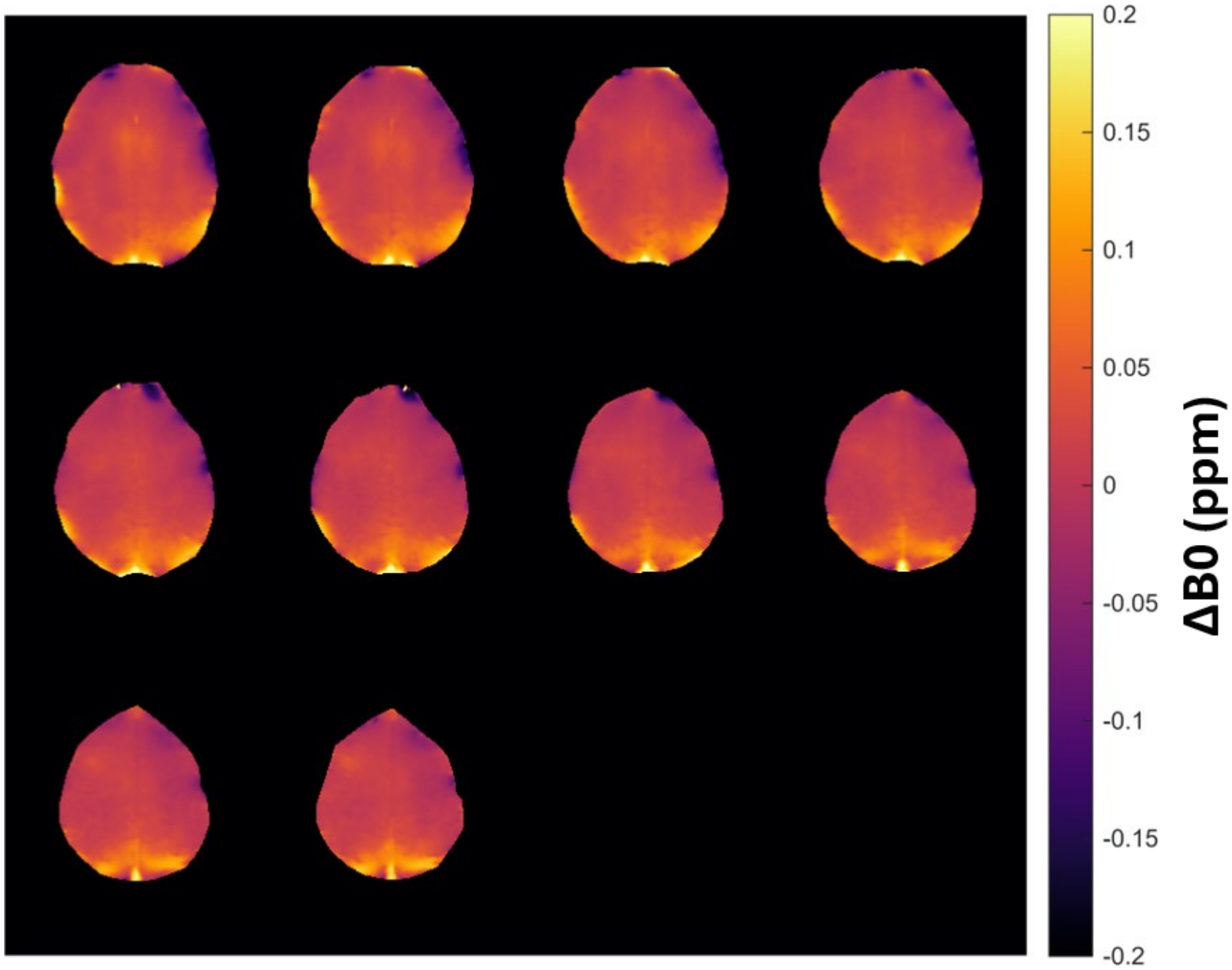

Supplemental Figure 3. Processed B_0 maps from a representative PwMS across all 10 slices acquired in the same position as the GluCEST acquisition.

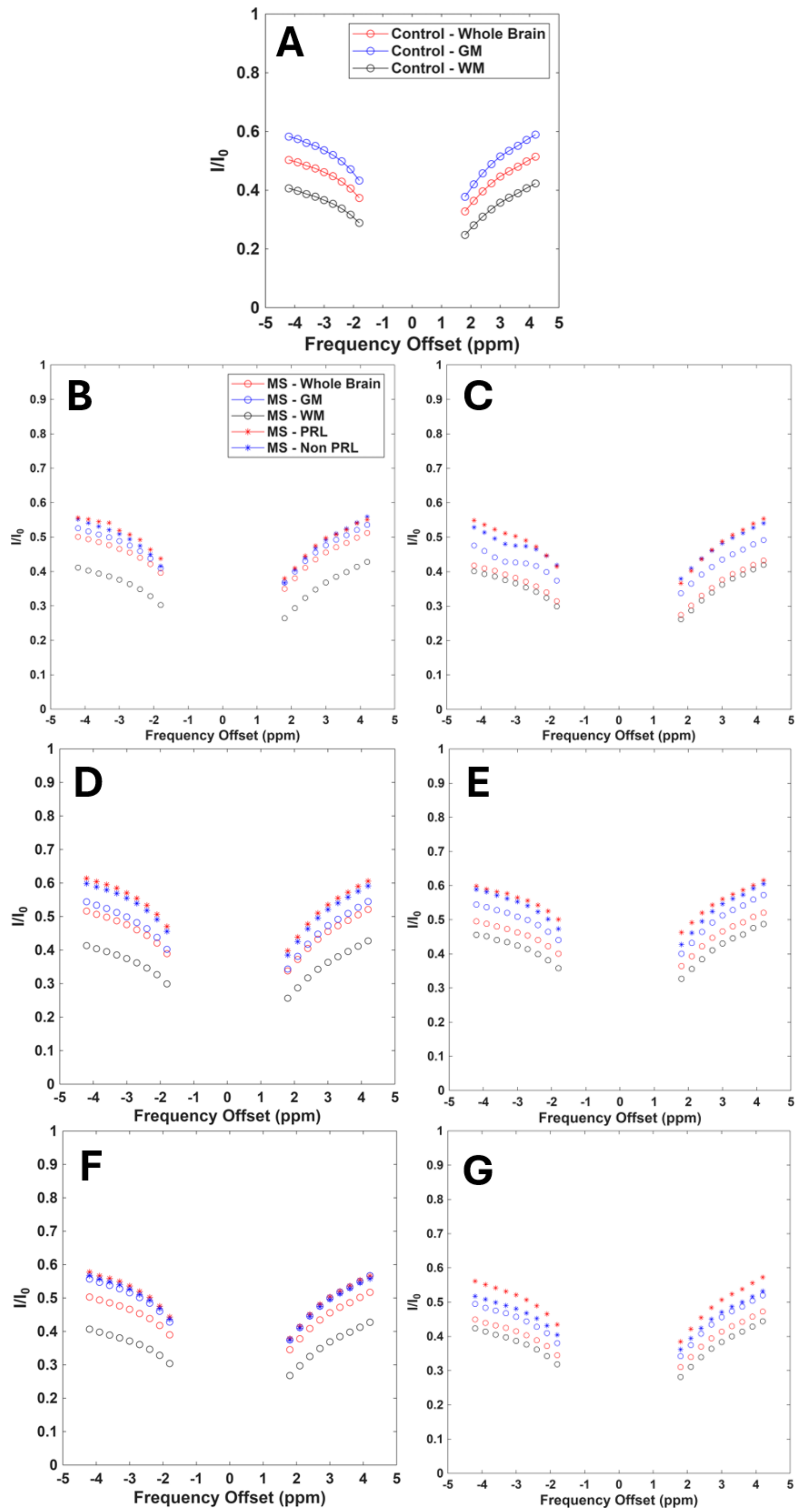

Supplemental Figure 4. Example representative partially acquired Z-spectra averaged over the relevant tissue regions between the healthy control subject (A) and MS participants 1 through 6 (B-G) (seen in figures 1 & 2).

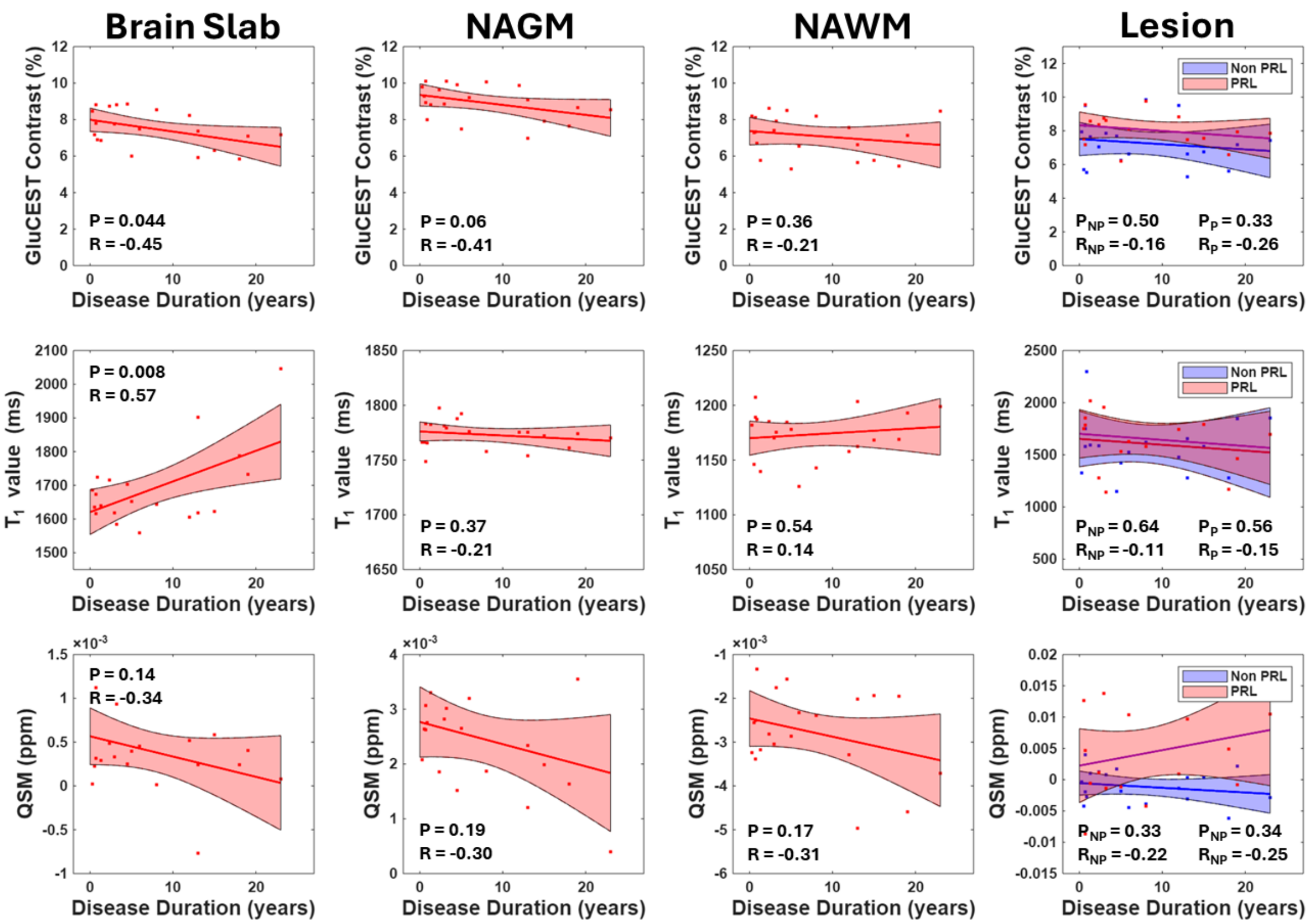

Supplemental Figure 5. A series of linear fit plots (including shaded CI region) with displayed P-values and Pearson's correlation coefficients for disease duration between MS GluCEST contrast, T_1 values, and QSM values. (**First Row**) GluCEST contrast values can be seen plotted with brain slab (*P* = 0.04, t-value = -2.16) having a significant negative correlation. (**Second Row**) T_1 values can be seen plotted with brain slab (*P* = 0.008, t-value = 2.93) having a significant positive correlation. (**Third Row**) QSM values can be seen plotted with no significant correlations observed. Segmented PRLs and non-PRLs indicated by the subscript P and NP respectively.

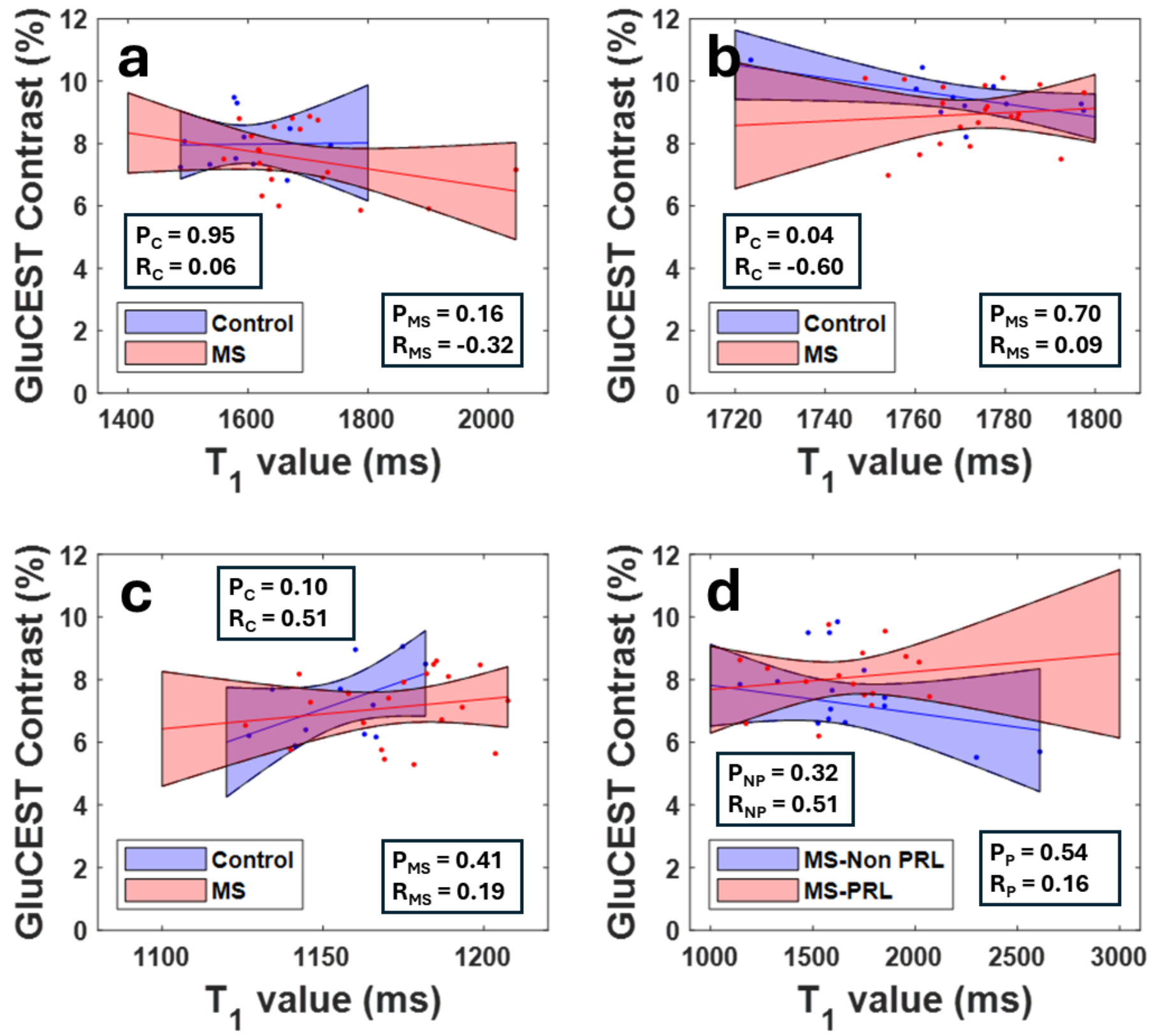

Supplemental Figure 6. Correlations between T_1 and GluCEST contrast values for each of the tissue regions including (a) whole brain slab, (b) gray matter, (c) white matter, and (d) PRL and Non PRL tissue. The only correlation that was observed was in healthy control diffuse gray matter tissue (P = 0.04, t-value = -2.28, R = -0.60).

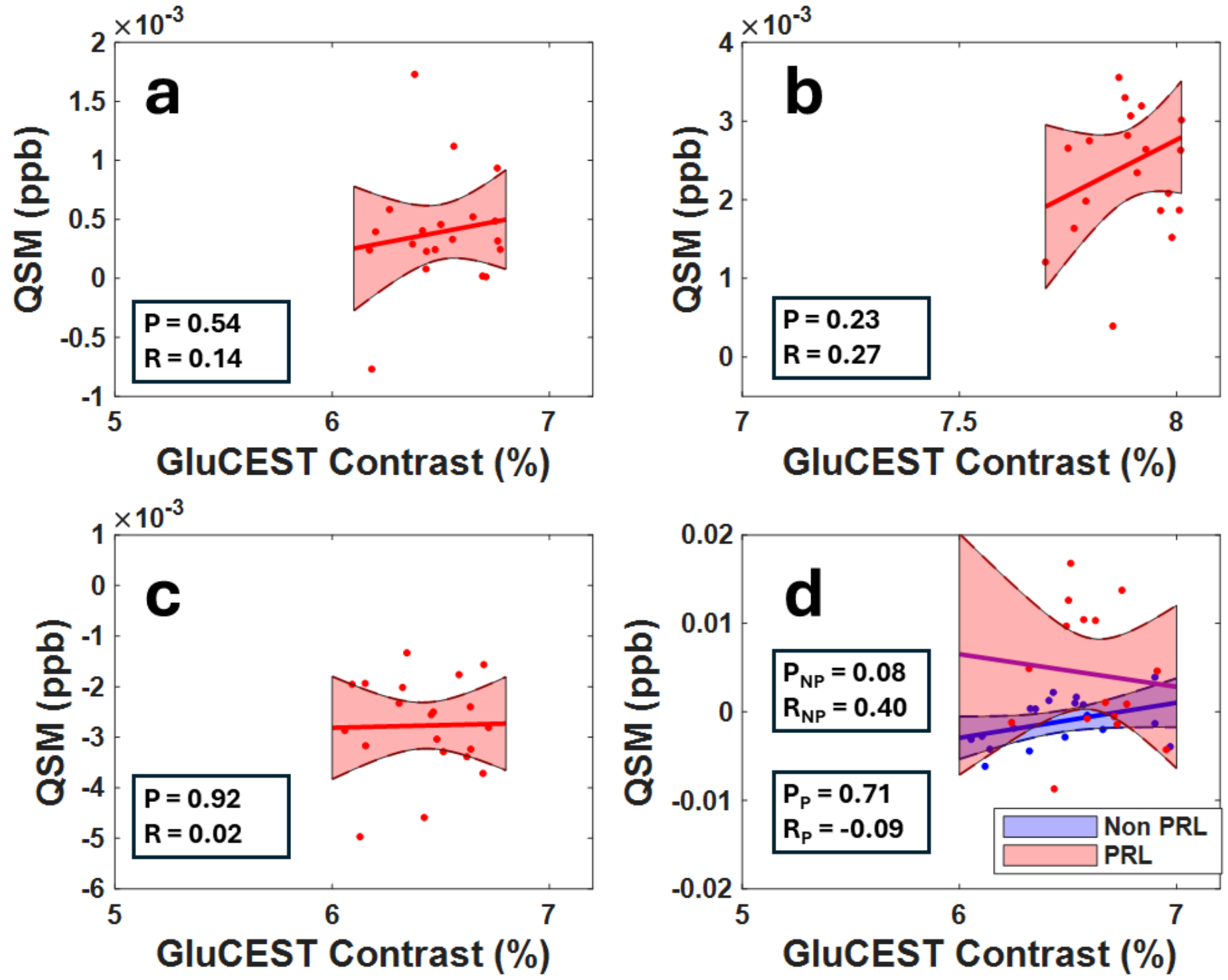

Supplemental Figure 7. Correlations between GluCEST contrast and QSM values for each of the tissue regions including (a) whole brain slab, (b) gray matter, (c) white matter, and (d) PRL and Non-PRL tissue. No correlations were observed in any of the tissue regions.
